# Prognostic value of the ascites characteristics in pseudomyxoma peritonei originating from the appendix

**DOI:** 10.3389/fsurg.2022.967296

**Published:** 2023-01-16

**Authors:** Bing Wang, Xibo Sun, Ruiqing Ma, Zhenpeng Yang, Huazhen Tang, Shuai Lu, Jinxiu Qu, Yuying Wang, Benqiang Rao, Hongbin Xu

**Affiliations:** ^1^ Department of Gastrointestinal Surgery, Beijing Shijitan Hospital, Capital Medical University, Beijing, China; ^2^ Key Laboratory of Cancer FSMP for State Market Regulation, Beijing, China; ^3^ Department of Breast Surgery, The Second Affiliated Hospital of Shandong First Medical University, Taian, China; ^4^ Department of Myxoma, Aerospace Center Hospital, Beijing, China

**Keywords:** pseudomyxoma peritonei, ascites characteristics, prognosis, overall survival, surgical oncology

## Abstract

**Background:**

Pseudomyxoma peritonei (PMP) is a rare disease, with the overall survival (OS) influenced by many factors. To date, no ascites characteristics have been reported to predict OS of patients with PMP. The present study therefore aims to describe the ascites characteristics for PMP and identify prognostic factors for survival.

**Methods:**

Between June 2010 and June 2020, 473 PMP patients who underwent cytoreductive surgery and hyperthermic intraperitoneal chemotherapy were included in a retrospective study. Survival analysis was performed with the Kaplan–Meier method by the log-rank test and a Cox proportional hazards model. Associations between categorical variables were analyzed using the chi-squared test.

**Results:**

Among all included patients, 61% were women. The median OS was 47 months (range, 4–124 months) at the last follow-up in December 2020. Ascites characteristics can be divided into light blood ascites, “Jelly” mucus ascites, and faint yellow and clear ascites. Multivariate Cox analysis showed that the degree of radical surgery, ascites characteristics, and pathological grade were independently associated with OS in PMP patients. The chi-squared test documented that faint yellow “Jelly” ascites were related to low-grade PMP and light blood ascites were associated with high-grade PMP (*P* < 0.01).

**Conclusions:**

Light blood ascites, incomplete cytoreduction surgery, and high-grade histopathology may predict poor OS in appendix-derived PMP.

## Introduction

Pseudomyxoma peritonei (PMP) is a rare disease characterized by the accumulation of ascites in the peritoneal cavity (1). It most commonly originates from a perforated epithelial tumor of the appendix (2). Patients begin to represent some clinical manifestations such as increased abdominal circumference, abdominal distension, and palpable masses. This eventually develops into malnutrition, bowel obstruction, and the like (3). The incidence rate was 3.2 people per million per year, and the prevalence rate was 22 people per million per year (4). Cytoreductive surgery (CRS) in combination with hyperthermic intraperitoneal chemotherapy (HIPEC) was the optimal treatment for PMP patients, as Sugarbaker clarified (5). The Peritoneal Surface Oncology Group International (PSOGI) recommended CRS and HIPEC as the standard treatment for PMP in 2014 (6). The application of previous systemic chemotherapy (PSC) has not yet been confirmed. Because of the availability of clinical data on patients receiving systemic chemotherapy prior to hospital admission, we analyzed the effect of PSC on postoperative survival in appendix-derived PMP using our database.

China has a large population base and a large number of PMP patients. Our center is one of the big centers for treating PMP in China. A previous study in our center showed that most patients in China could not obtain a correct PMP diagnosis in a timely manner (7), and most of the patients were in the middle and late stages of the disease. Patients exhibited different features of the ascites. Long-term treatment experience tells us that the characteristics of patients with ascites can be expressed as light blood ascites, “Jelly” mucus ascites, and faint yellow and clear ascites. However, there are no reports on the characteristics of ascites until now. In the present study, we intended to evaluate the prognostic value of ascites characteristics in patients with PMP. It is innovative compared with the previous studies.

## Patients and methods

### Study population

All data for this retrospective study were reviewed from a follow-up database of the Aerospace Center Hospital, the largest single center treating PMP in China. The study was approved by the institutional review board (IRB) of the Aerospace Center Hospital, and all participants signed informed consent before operation.

The study acquired 473 patients with a PMP diagnosis between June 2010 and June 2020. Inclusion criteria are as follows: (1) diagnosis of appendix-derived PMP on histology and histopathologic subtype confirmed by two experienced pathologists; and (2) treatment with CRS and HIPEC. Exclusion criteria are as follows: (1) PMP derived from other organs (e.g., ovary, colon, urachus, pancreas, biliary tract, and intestinal duplication); (2) incomplete medical records; and (3) loss to follow-up.

### CRS and HIPEC

We used a median incision entering the abdominal cavity and then collected the ascites and assessed the peritoneal cancer index (PCI). The PCI evaluated intraoperatively in each of the 13 abdominopelvic regions (nine anatomical regions in the abdomen and four segments in the small bowel) was scored on a scale from 0 to 3 and summed (8). The CRS was in accordance with the standard operation method (9). After CRS, the completeness of cytoreduction (CCR) was scored, where CCR 0 indicated no visible residual tumors and CCR 1 indicated residual tumors of less than 2.5 mm. Any residual tumor nodules between 2.5 mm and 2.5 cm were labeled as CCR 2, whereas residual tumor nodules larger than 2.5 cm were labeled as CCR 3 (5). CCR 0 and CCR 1 were considered as performing complete CRS (CCRS). For patients who cannot achieve CCRS, maximum tumor debulking (MTD) was performed. CCR 2 and CCR 3 were considered as having undergone MTD (5), and CCR2 and CCR3 outcomes were considered examples of incomplete cytoreduction surgery (10).

Intraoperative HIPEC was delivered in all included patients for 60 min using a closed-abdomen technique with mitomycin (20 mg/m^2^) and a HIPEC machine that was heated to 43 °C, maintaining the intra-abdominal temperature.

### Study parameters

The study included the following clinicopathological parameters: gender, age at hospitalization, status, overall survival (OS), history of (with/without) PSC, prior surgical score (PSS), intraoperative PCI, CCR, ascites volume, ascites characteristics, history of (with/without) lymphatic metastasis, pathological grade, and follow-up time.

PSS was scored from 0 to 3 among these variables, where PSS 0 indicated no surgery or biopsy had been performed for the tumor, PSS 1 indicated surgery had been conducted in one abdominal region, PSS 2 indicated surgery had been done in two to five regions, and PSS 3 indicated surgery had been carried out in more than five regions (11).

According to the 2016 PSOGI criteria, the pathological diagnosis was classified into four categories, acellular mucin (AC), low-grade mucinous carcinoma peritonei (LG-MCP), high-grade mucinous carcinoma peritonei (HG-MCP), or high-grade mucinous carcinoma peritonei with signet ring cells (HGMC-S) (12). Patients with AC were excluded due to incomplete data.

### Statistical analysis and follow-up

Statistical analyses were conducted using SPSS 20.0 (SPSS, Chicago, Illinois, United States). Continuous data were presented as medians and ranges. Categorical data were presented as numbers and percentages. Univariate survival analysis was performed with the Kaplan–Meier method and the log-rank test. All variables were included in the multivariate analysis, which used a Cox proportional hazards model to identify independent prognostic factors for survival. Categorical variables were analyzed using the chi-square test. All live patients were censored. *P* < 0.05 was considered statistically significant.

Follow-up was done via a telephone and or re-examination. The follow-up time was from the first operation date to December 2020, and the OS was counted. All included patients were followed up.

## Results

### Clinicopathological characteristics

The clinicopathological features of the 473 included patients are presented in Table 1. The majority of the patients were women (61%), and the median age at hospitalization was 58 years (range, 26–85 years). There were 126 (27%) patients who underwent systemic chemotherapy before surgery, and PSS was 0/1 and 2/3 in 244 (52%) and 229 (48%) patients, respectively. The median intraoperative PCI score was 29 (range, 2–39 scores), and the intraoperative PCI score was <25 and ≥25 in 120 (25%) and 353 (75%) patients, respectively. The CCR score was 0/1 and 2/3 in 222 (47%) and 251 (53%) patients, respectively.

**Table 1:** Patients’ clinical and demographic data.

Characteristics	No. of patients
Gender	
Male	186 (39%)
Female	287 (61%)
Age at hospitalization (years)	
Median (range)	58 (26–85)
<50	103 (22%)
≥50	370 (78%)
PSC	
Yes	126 (27%)
No	347 (73%)
PSS	
0/1	244 (52%)
2/3	229 (48%)
Preoperative PCI	
Median (range)	29 (2–39)
<25	120 (25%)
≥25	353 (75%)
CCR	
Median (range)	2 (0–3)
0/1	222 (47%)
2/3	251 (53%)
Ascites volume (ml)	
Median (range)	3,000 (0–20,000)
<3,000	227 (48%)
≥3,000	246 (52%)
Ascites characteristics	
Light blood	141 (30%)
“Jelly” mucus	191 (40%)
Faint yellow and clear	127 (27%)
No ascites	14 (3%)
Lymphatic metastasis	
Yes	26 (5%)
No	447 (95%)
Pathological grade	
LG-MCP	328 (69%)
HG-MCP	99 (21%)
HGMC-S	46 (10%)

PSC, previous systemic chemotherapy; PSS, prior surgical score; PCI, peritoneal cancer index; CCR, completeness of cytoreduction; LG-MCP, low-grade mucinous carcinoma peritonei; HG-MCP, high-grade mucinous carcinoma peritonei; HGMC-S, high-grade mucinous carcinoma peritonei with signet ring cells.

The median ascites volume of included patients was 3,000 ml (range, 0–20,000 ml). The Ascites volume was <3,000 and ≥3,000 in 227 (48%) and 246 (52%) patients, respectively. A total of 141 (30%) patients presented as light blood ascites, 191 (40%) patients presented as “Jelly” mucus ascites, 127 (27%) patients presented as faint yellow and clear ascites, and 14 (3%) patients had no ascites. Twenty-six (5%) patients had lymphatic metastasis. Pathological diagnosis showed that 328 (69%) patients had LG-MCP, 99 (21%) patients had HG-MCP, and 46 (10%) patients had HGMC-S.

### Survival analysis

**Figure 1:**
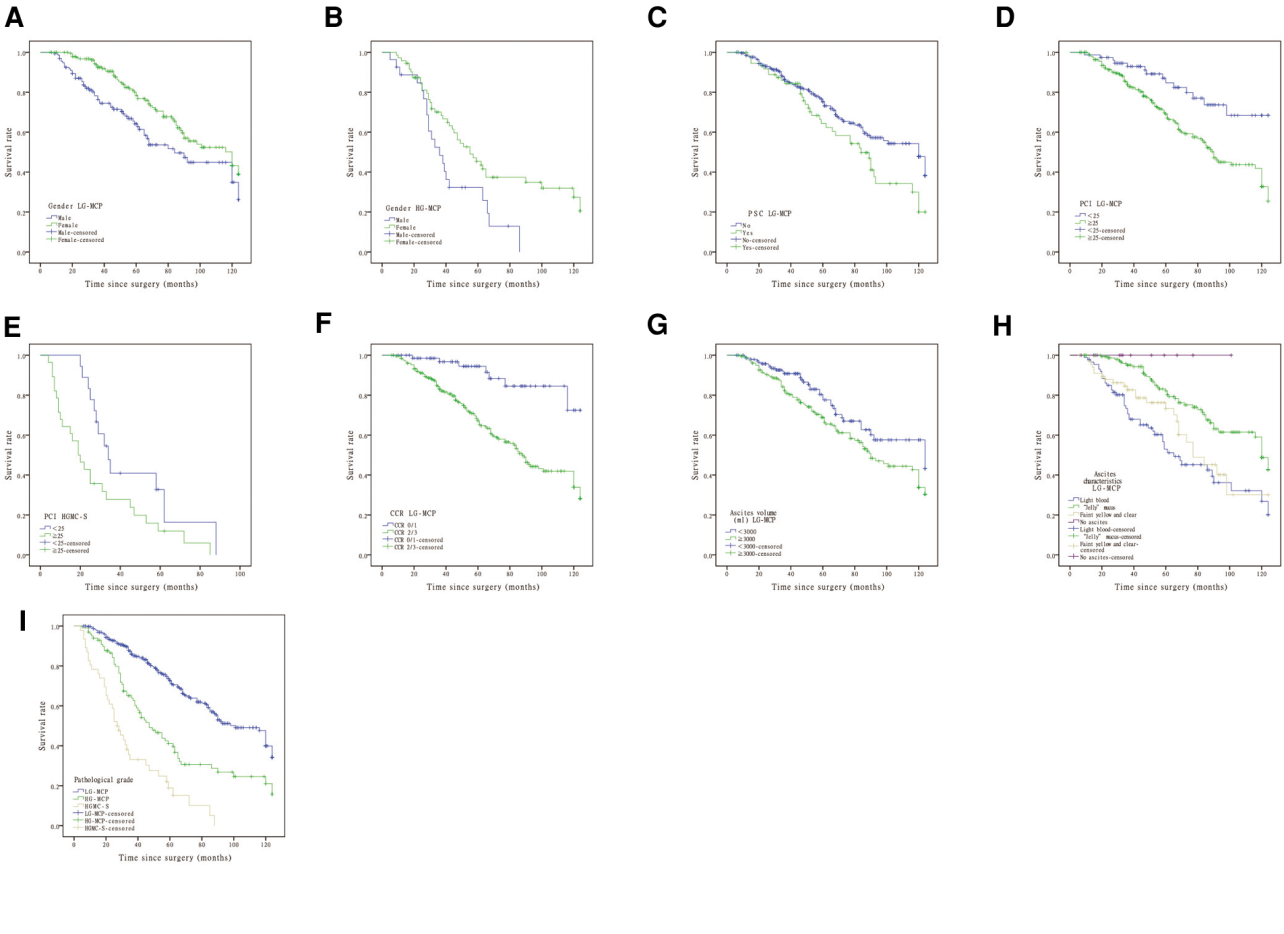
Survival curves for (**A**) gender of LG-MCP, (**B**) gender of HG-MCP, (**C**) PSC of LG-MCP, (**D**) PCI of LG-MCP, (**E**) PCI of HGMC-S, (**F**) CCR of LG-MCP, (**G**) ascites volume of LG-MCP, (**H**) ascites characteristics of LG-MCP, and (**I**) pathological grade. LG-MCP, low-grade mucinous carcinoma peritonei; HG-MCP, high-grade mucinous carcinoma peritonei; PSC, previous systemic chemotherapy; PCI, peritoneal cancer index; HGMC-S, high-grade mucinous carcinoma peritonei with signet ring cells; CCR, completeness of cytoreduction.

Prognostic factors for OS on univariate and multivariate analyses are presented in Tables 2 and 3. The median OS was 47 months (range, 4–124 months) at the last follow-up in December 2020. Univariate analysis showed that gender was statistically different in LG-MCP and HG-MCP. Patients who received preoperative PSC may predict poor survival in LG-MCP. Intraoperative PCI ≥25 can indicate poor survival in LG-MCP and HGMC-S. The degree of cytoreduction reaching CCR 0 and the volume of ascites <3,000 ml can predict better survival in LG-MCP patients. Patients stratified by ascites characteristics of PMP (light blood and “Jelly” mucus and faint yellow and clear and no ascites) were prognostic for OS by the log-rank test in patients with LG-MCP. Among all included patients, the pathological grade of PMP (LG-MCP vs. HG-MCP vs. HGMC-S) was prognostic for OS by the log-rank test (*P* = 0.000) (Figure 1). After the Cox proportional hazards regression analysis, CCR 2/3, light blood ascites, and high pathological grade were independently associated with poor OS in PMP patients.

**Table 2:** Univariate analysis affecting overall survival after CRS.

Variables	Log-rank *P* value of univariate analysis			
	Overall	LG-MCP	HG-MCP	HGMC-S
	*P* value	*P* value	*P* value	*P* value
Gender (male vs. female)	0.000*	0.003*	0.012*	0.652
Age (<50 vs. ≥ 50, years)	0.980	0.546	0.748	0.708
PSC (yes vs. no)	0.003*	0.025*	0.964	0.926
PSS (0/1 vs. 2/3)	0.125	0.063	0.062	0.396
Intraoperative PCI (<25 vs. ≥ 25)	0.022*	0.001*	0.528	0.024*
CCR (0/1 vs. 2/3)	0.000*	0.000*	0.528	0.057
Ascites volume (<3,000 vs. ≥ 3,000)	0.126	0.029*	0.521	0.056
Ascites characteristics (light blood vs. “Jelly” mucus vs. faint yellow and clear vs. no ascites)	0.000*	0.000*	0.166	0.784
Lymphatic metastasis (yes vs. no)	0.265	0.425	0.073	0.462
Pathological grade (LG-MCP vs. HG-MCP vs. HGMC-S)	0.000*			

CRS, cytoreductive surgery; PSC, previous systemic chemotherapy; PSS, prior surgical score; PCI, peritoneal cancer index; CCR, completeness of cytoreduction; LG-MCP, low-grade mucinous carcinoma peritonei; HG-MCP, high-grade mucinous carcinoma peritonei; HGMC-S, high-grade mucinous carcinoma peritonei with signet ring cells.

* *P* < 0.05.

**Table 3:** Multivariate analysis affecting overall survival.

Variables	*B*	SE	Wald	*P* value	Exp (B)	95% CI for Exp (B)	
						Bottom	Upper
Gender	−0.218	0.167	1.695	0.193	0.804	0.580	1.116
Age	0.006	0.007	0.824	0.364	1.006	0.993	1.020
PSC	0.283	0.157	3.253	0.071	1.327	0.976	1.805
PSS	−0.184	0.096	3.683	0.055	0.832	0.689	1.004
Preoperative PCI	0.009	0.014	0.427	0.513	1.009	0.982	1.037
CCR	0.326	0.125	6.780	0.009	1.386	1.084	1.772
Ascites volume	0.000	0.000	1.660	0.198	1.000	1.000	1.000
Ascites characteristics	−0.216	0.072	8.958	0.003	0.806	0.700	0.928
Lymphatic metastasis	−0.220	0.276	0.634	0.426	0.803	0.467	1.379
Pathological grade	0.882	0.104	72.524	0.000	2.416	1.972	2.960

PSC, previous systemic chemotherapy; PSS, prior surgical score; PCI, peritoneal cancer index; CCR, completeness of cytoreduction; B, unstandardized coefficient; SE, standard error; Exp, odds ratio; CI, confidence interval.

### Relationship between ascites characteristics and pathological grade

To analyze the relationship between ascites characteristics and pathological grade, we used the chi-square test to verify the hypothesis. The results documented that faint yellow “Jelly” ascites was related to low-grade PMP and light blood ascites was associated with high-grade PMP (*P* < 0.01) (Table 4).

**Table 4:** Relationship between ascites characteristics and pathological grade.

Variables	LG-MCP	HG-MCP	HGMC-S
Light blood	36	88	17
“Jelly” mucus	159	28	4
Faint yellow and clear	69	34	24

LG-MCP, low-grade mucinous carcinoma peritonei; HG-MCP, high-grade mucinous carcinoma peritonei; HGMC-S, high-grade mucinous carcinoma peritonei with signet ring cells.

χ^2^ test, *P* < 0.01.

## Discussion

Patients with pseudomyxoma peritonei are often accompanied by a large volume of ascites during the development of the disease, and the characteristics of ascites are various. To the best of our knowledge, the current study is the first to report the ascites characteristics to predict prognosis for PMP patients. In this research, ascites was divided into light blood ascites, “Jelly” mucus ascites, and faint yellow and clear ascites. The prognostic value showed good performance.

The advantage of the present study is that the missing follow-up rate was very low, with only eight patients lost to follow-up. Because we had arranged for a physician to be responsible for follow-up at the beginning of the establishment of our follow-up database, this ensured the reliability of the research concluded with a low level of missed follow-up visits.

In the present study, in univariate analysis, gender was prognostic for OS in patients with LG-MCP and HG-MCP. In a previous study in PMP, the male gender (HR, 1.61) had worse OS (13). Another study showed that the female gender (*p* = 0.045) was associated with improved OS (14), which was consistent with our study. The most likely reason is that women tend to present at an earlier stage than men, which may be related to rapidly enlarging ovarian masses and clinical symptoms. A previous study had confirmed that suspected ovarian tumor was the most common cause for surgery among women PMP patients (15). Men often present at an advanced stage because the disease is initially asymptomatic (1).

In the present series on the univariate analysis, the OS of LG-MCP patients with PSC appeared lower than the OS of those without PSC. It seems interesting compared with other diseases (16–18). A selection bias could have occurred in this article, as a small number of LG-MCP patients received PSC. Tumor biology may have played a role in the setting because suggestion has demonstrated that patients with aggressive diseases received systemic treatment. Our finding further validates this notion. A recent study revealed that OS in the non-PSC group was significantly better than that in the PSC group for low-grade PMP, although no difference was found in high-grade PMP (19). Consistent with our study, one previous report suggested that PSC was correlated to worse OS (20). Another study indicated that no PSC was an independent predictor of a better OS using multivariate analysis (21). In 2012, the Morris team published a multicenter large sample study with 2,298 PMP patients. The result showed that PSC was an independent risk factor for a poorer OS (11). In China, many patients did not undergo standardized cytoreduction for the first time. Patients who come to our center are often accompanied by a large tumor burden, which leads to a relatively high tumor burden and low radical rate. However, our data showed that patients had better survival after receiving standardized CRS and HIPEC.

CRS and HIPEC are regarded as standard treatments for PMP. Our study further confirmed the impact of CCR and pathological grade on prognosis. This is not surprising because many reports are compatible with it (22–25). Tumor burden plays an important role in predicting the prognosis. Findings showed that a large tumor burden might have poor survival, especially in LG-MCP. Consistent with this, one previous report showed that PCI > 22 correlated with prognosis in PMP (26). However, in our study, accurate intraoperative assessment of PCI can be used to predict prognosis, which is innovative compared with the previous studies.

In China, a majority of patients are already accompanied by a large volume of ascites at the time of consultation in our center, for the reasons that PMP patients were not diagnosed in a timely manner or a lack of concept for treatment. However, there is no report on the features of ascites. It is novel compared with the other reports. We found that ascites volume over 3,000 ml may predict poor survival in LG-MCP, and no ascites was associated with high survival. We further analyzed the potential relationship between ascites characteristics and pathological grade. The results revealed that light blood ascites was associated with high pathological grade, while clear mucinous ascites was related to low pathological grade. In this setting among multivariate analysis, ascites characteristic was an independent prognostic factor for OS. In a previous study, Kanayama et al. determined the role of vascular endothelial growth factor (VEGF) in the progression in nude mice. They observed that tumors produced hyperpermeability of peritoneal blood vessels, bloody ascites, and short survival time (27). Lymphatic metastasis had no impact on OS due to its limited clinical data.

There were several limitations to the present study. First, due to the limitations of the retrospective study design, some data were not complete in the database. Second, owing to the small number of patients with AC and incomplete data, these patients were not included in the study. Finally, some other prognostic factors were not included in the study, such as GNAS mutation, which has been proven to be a significantly shorter median progression-free survival in PMP patients (28). Future work will further confirm this hypothesis.

## Data Availability

The original contributions presented in the study are included in the article/Supplementary Material, further inquiries can be directed to the corresponding author.
